# Rethinking corticosteroids use in oncology

**DOI:** 10.3389/fphar.2025.1551111

**Published:** 2025-03-26

**Authors:** Pierrick Martinez, Jean-Marc Sabatier

**Affiliations:** ^1^ Association Cancer et Métabolisme, Nîmes, France; ^2^ Institut de NeuroPhysiopathologie (INP), CNRS UMR 7051, Marseille, France

**Keywords:** corticosteroids, glucocorticoids, glycolysis, glutaminolysis, renin-angiotensin system, vitamin D, microbiome

## Abstract

Corticosteroids (CSs), widely used in oncology for their anti-inflammatory and immunosuppressive properties, help manage cancer-related symptoms and side effects. However, their long-term use may negatively affect patient survival and exacerbate tumor progression. Elevated glucose and glutamine metabolism, disruption of vitamin D levels, and alterations in the microbiome are some of the key factors contributing to these adverse outcomes. Approaches such as ketogenic diets, fasting, sartans, and vitamin D supplementation have shown promise in providing similar benefits to CSs while mitigating the risks associated with the mechanisms identified as contributing to tumor progression. This perspective underscores the necessity for a reevaluation of CSs use in cancer care and advocates for further research into safer, more effective therapeutic strategies.

## 1 Introduction

Corticosteroids (CSs) are synthetic drugs with a broad range of effects on cells and tissues, widely used in oncology due to their ability to regulate inflammation and modulate the immune response (Goodman et al., 2023). These include hydrocortisone, prednisone, prednisolone, methylprednisolone, and dexamethasone (Faggiano et al., 2022). While hydrocortisone has both glucocorticoid (anti-inflammatory and immunosuppressive) and mineralocorticoid (electrolyte balance) activities, the other CSs mentioned, including prednisone, prednisolone, methylprednisolone, and dexamethasone, exhibit little to no mineralocorticoid activity. In oncology, high-dose CSs are commonly used for a variety of purposes: managing cancer-related symptoms such as fatigue, shortness of breath, pain from bone metastases, or cerebral edema caused by brain metastases; reducing side effects of cancer treatments; managing oncological emergencies; enhancing anti-cancer effects, particularly in hematologic cancers; and treating comorbidities like autoimmune diseases (Faggiano et al., 2022). In this review, we will explore the effects of CSs on survival and the long-term side effects associated with their prolonged use. We will then investigate the mechanisms by which CSs may influence tumor progression. Four key mechanisms have been identified that could explain the negative impact of CSs on survival: altered cancer cell metabolism, the renin-angiotensin system (RAS), vitamin D metabolism, and the microbiome. Based on these mechanisms, we propose therapeutic strategies that may complement or potentially replace CSs in oncological care.

## 2 Corticosteroids use in oncology: impact on survival, risks, and potential adverse effects

CSs have been widely used in oncology for decades, often as part of standardized treatment protocols for various hematologic and solid malignancies. However, as monotherapy, CSs have not been able to induce long-lasting remissions, let alone cures (Pearson and Eliel, 1950). Their primary role has been in symptom management and as adjuncts to other therapies, rather than as standalone curative agents. In this section, we will examine the role of CSs in oncology, assessing their true impact on treatment outcomes, their contribution to observed survival benefits, and the risks associated with prolonged exposure.

### 2.1 Effect on survival in hematological cancers

In Non-Hodgkin Lymphoma (NHL), individuals with relapsed or refractory large B-cell lymphoma (LBCL) treated with axi-cel showed an association between higher cumulative corticosteroid doses and shorter progression-free survival (PFS) and overall survival (OS) particularly when corticosteroids were used early or for a prolonged duration (Strati et al., 2021). Furthermore, alternative chemotherapy regimens have shown promising results without CSs. For instance, the bendamustine + rituximab (B + R) regimen, which does not contain CSs, achieved a significantly longer PFS (69.5 vs. 31.2 months; p < 0.0001) and demonstrated better tolerability compared to Rituximab + Cyclophosphamide + Doxorubicine + Vincristine + Prednisone (R-CHOP). However, this improvement in PFS did not translate into a significant overall survival benefit. These findings support B + R as a preferred first-line treatment option for patients with previously untreated indolent lymphoma (Rummel et al., 2013). In Diffuse Large B-Cell Lymphoma (DLBCL), recent evidence also challenges the necessity of CSs. Chimeric Antigen Receptor T-cell (CAR-T cell) therapy has shown higher response rates compared to various alternative chemotherapy regimens, including Cyclophosphamide + Doxorubicine + Vincristine + Prednisone (CHOP, with CSs) (Sermer et al., 2020). Additionally, the ZUMA-1 trial demonstrated that the CAR-T therapy axicabtagene ciloleucel (73% without CSs) provided durable responses in patients with relapsed or refractory large B-cell lymphoma, after failure of multiple prior therapies, including R-CHOP with CSs (Neelapu et al., 2017). A similar trend is observed in Hodgkin Lymphoma (HL). Studies comparing Mustargen (mechlorethamine) + Oncovin (vincristine) + Procarbazine + Prednisone (MOPP, which includes prednisone) and Adriamycine + Bléomycine + Vinblastine + Dacarbazine (ABVD, which does not) indicate that ABVD achieves superior efficacy in terms of complete response rate and failure-free survival, with fewer hematologic toxicities. However, overall survival at 5 years did not differ significantly between the treatment groups (Canellos et al., 1992). Alternating MOPP/ABVD regimens remained similar to ABVD monotherapy (Duggan et al., 2003). Another study found no significant difference in survival between MOPP and ABVD (Ng et al., 2004). The role of CSs in multiple myeloma (MM) can also be questioned. An analysis of 18 clinical trials found no significant difference in overall efficacy between melphalan + prednisone (M + P) and combination chemotherapy (CCT) (Gregory et al., 1992) M+P was more effective in patients with good prognosis (P = .02), while CCT tended to be better for those with poor prognosis (P = .07) (Gregory, Richards et al. 1992). More recent studies suggest that adding CSs to lenalidomide maintenance may not provide significant survival benefits (Bringhen et al., 2017; Bonello et al., 2019). Collectively, these findings suggest that while CSs have historically played a key role in lymphoid malignancies, they are not always indispensable. Their use should be carefully reassessed on a case-by-case basis, weighing potential benefits against toxicity risks. The only cancer type where their necessity could remain unquestioned is leukemia, particularly acute lymphoblastic leukemia (ALL) (Pourhassan et al., 2024). Even in leukemia, certain clinical situations could justify exploring alternative therapeutic approaches. Glucocorticoid resistance in ALL is a major challenge that compromises treatment efficacy (Olivas-Aguirre et al., 2021). Additionally, recent research on CAR T-cell therapy reported that 30 patients received CAR-T anti-CD19. Among them, nine received tocilizumab to manage cytokine release syndrome (CRS), and six required additional CSs. Most patients were successfully treated without the need for CSs (Maude et al., 2014). This further suggests that even in ALL, alternative strategies could be viable. This assumption needs further validation, as the widespread standardization of CSs in this setting has limited the availability of comparative data. More studies are required to evaluate their true indispensability.

### 2.2 Effect on survival in solid tumors

In the case of solid tumors, their use is more uncertain and may be either beneficial or detrimental. The administration of dexamethasone in glioblastoma, at the start of radiotherapy is associated with reduced median survival and poorer clinical outcomes. Patients on steroids had significantly lower OS (12 vs. 17 months) and PFS (5.3 vs. 6.4 months) (Pitter et al., 2016). The negative effects of steroids were especially pronounced in patients who received radiotherapy alone. These effects were less pronounced, or even non-significant, in those treated with combined chemotherapy and radiotherapy (Pitter et al., 2016). This is confirmed in a meta-analysis of 22 studies including 8,752 patients, which showed that the use of CSs in patients with glioblastoma is associated with a significant reduction in OS (HR = 1.54; 95% CI: 1.37-1.75; p < 0.01) and PFS (HR = 1.28; 95% CI: 1.1-1.49; p < 0.01) (Petrelli et al., 2021). A recent meta-analysis, which included 76 studies and a total of 83,614 patients, revealed that CSs use in advanced solid cancers is associated with decreased OS (hazard ratio [HR] = 1.18, 95% confidence interval [CI]: 1.10–1.26; P < .01, based on 69 studies). PFS was also negatively impacted in steroid users compared to non-users (HR = 1.13, 95% CI: 1.01–1.26; P = .03, based on 40 studies). The study analyzed the effects of steroids across various solid tumors, including non-small cell lung cancer (NSCLC), prostate cancer, breast cancer, gastrointestinal cancers, melanoma, pancreatic cancer, ovarian cancer, and Kaposi’s sarcoma. Steroid use was evaluated in different settings, such as palliative care, in combination with immunotherapies, chemotherapies, and hormonal treatments, as well as post-surgical management (Petrelli et al., 2020). Similarly, a recent systematic review and meta-analysis of 4,045 patients found that steroid use in individuals undergoing immune checkpoint inhibitor (ICI) therapy increased the risk of disease progression and death by 34% and 54% respectively, compared to non-steroid users (Petrelli et al., 2020). Additionally, another meta-analysis demonstrated that CSs administration for cancer-related indications was significantly associated with worse PFS (HR = 1.735, 95% CI: 1.381–2.180) and OS (HR = 1.936, 95% CI: 1.587–2.361) in ICI-treated patients (Wang et al., 2021).

### 2.3 Adverse effects of corticosteroids

Their use is associated with numerous side effects affecting various systems. Metabolically, they can lead to hyperglycemia, hypertension and weight gain (Kulkarni et al., 2022). Endocrinologically, they increase the risk of Cushing’s syndrome (Oray et al., 2016) and may cause adrenal insufficiency (Caplan et al., 2017). Gastrointestinally, CSs promote the development of gastritis, peptic ulcers, and increase the risk of gastrointestinal bleeding, particularly when combined with nonsteroidal anti-inflammatory drugs (NSAIDs) (Caplan et al., 2017). Cardiovascularly, they raise blood pressure and the risk of heart disease (Pimenta et al., 2012). The risk of hypertension is increased by ∼2-fold in patients treated with CSs regardless of treatment duration (Wei et al., 2004). Furthermore, it has been reported that the use of CSs increases the risk of coronary heart disease, ischemic heart disease, heart failure and even sudden death (Wei et al., 2004). The ALL AIEOP/BFM 2000 study demonstrated a significant increase in deaths during induction therapy with dexamethasone (10 mg/m^2^), mainly due to severe bacterial and fungal infections, particularly in patients aged ≥10 years (4.5% vs. 2.4% with prednisone) (Schrappe et al., 2008).

Although CSs are effective in symptom management, their long-term survival benefits remain questionable. Given their potential long-term adverse effects, they are not recommended as a prolonged treatment for survival improvement. However, their short-term use can be beneficial in urgent oncological situations such as spinal cord compression, and superior vena cava syndrome, where they play a crucial role in reducing inflammation and temporarily alleviating symptoms (Faggiano et al., 2022).

## 3 Impact of corticosteroids on cancer cells: unraveling tumor progression and systemic risks

CSs influence various metabolic processes, including glucose and glutamine metabolism, the renin-angiotensin system, vitamin D metabolism, and the microbiome. While these drugs are often considered from the perspective of their beneficial effects, their impact on cancer progression and the systemic balance of patients must be better understood to optimize their use and limit their harmful effects. This section explores these mechanisms to highlight the associated risks and the need for alternative approaches.

### 3.1 Cancer cell metabolism: a therapeutic contradiction

The metabolism of cancer cells relies heavily on glucose and glutamine as their primary energy sources, and an increase in glucose and glutamine levels may enhance malignancy (Seyfried et al., 2020). The “Warburg effect,” described the last century (Warburg, 1956), highlights the critical role of glucose, while the recent recognition of glutamine’s role has reinforced the idea that both substrates are crucial for tumor growth (Seyfried et al., 2020; Lee et al., 2024). However, CSs use leads to a marked increase in blood glucose levels. A study by Limbachia et al. (2024) demonstrated that all CSs increase glucose concentrations, but dexamethasone and methylprednisolone induce more significant spikes compared to prednisolone or hydrocortisone. Consequently, 10%–30% of cancer patients experience acute hyperglycemia episodes (Hwangbo and Lee, 2017), and some develop corticosteroid-induced diabetes (Suh and Park, 2017). Notably, one study found that 20% of non-diabetic patients treated with dexamethasone for gastrointestinal cancer developed steroid-induced diabetes (Jeong et al., 2016). These metabolic effects have direct clinical consequences: a meta-analysis of 2,168 patients revealed that hyperglycemia, whether pre-existing or corticosteroid-induced, is associated with a significant reduction in overall survival in brain cancers (Liu et al., 2016). Similarly, increased glutamine levels under CSs treatment (Thibault et al., 2008), due to the activation of glutamine synthetase (Kazazoglou et al., 2021) and glutaminase (Sarantos et al., 1992), may promote tumor proliferation.

These findings raise a critical question: is the routine use of CSs, particularly in palliative care patients, truly justified when fatigue remains one of the most prevalent symptoms? While CSs are known to modulate inflammation and improve certain aspects of quality of life, their metabolic effects could paradoxically exacerbate fatigue rather than alleviate. Moreover, their impact on glucose and glutamine metabolism presents a therapeutic contradiction. By increasing circulating glucose and glutamine levels—key fuels for tumor growth—CSs might unintentionally accelerate disease progression. This duality underscores the complexity of their use in oncology, highlighting the need for a more nuanced approach that weighs their symptomatic benefits against potential long-term oncologic risks.

### 3.2 Renin-angiotensin system: an amplifying mechanism

The RAS plays a key role in maintaining systemic adaptation in cancer (Martinez and Sabatier, 2025). The renin-angiotensin system (RAS) is initiated when the liver releases angiotensinogen, a precursor protein that is cleaved by renin to generate angiotensin I, an inactive form. Angiotensin I is subsequently converted into angiotensin II (Ang II) by the angiotensin-converting enzyme (ACE), which is primarily produced in the liver and functions mainly in the lungs. Ang II exerts its physiological effects through two main receptor types: angiotensin type 1 receptor (AT1R) and type 2 receptor (AT2R) (Catarata et al., 2020). AT1R is frequently overexpressed on the cell surface in many types of cancer (Roth et al., 2019), where it promotes proangiogenic and proinflammatory processes. The use of CSs has been linked to an increase in ACE levels (Fishel et al., 1995), which leads to elevated Ang II concentrations and enhanced expression of AT1R (Shelat et al., 1999). A study highlights that the overexpression of the AT1R receptor is associated with poor overall survival in patients with ESCC and that the angiotensin II/AT1R signaling pathway promotes tumor growth, partly through mTOR activation (Li et al., 2016). The impact of CSs on the renin-angiotensin system, by increasing the expression of angiotensin II and its AT1R receptor, could enhance pro-angiogenic and pro-inflammatory processes, thereby promoting tumor progression and metastasis.

### 3.3 Vitamin D metabolism: an underestimated impact?

CSs also alter vitamin D metabolism, a hormone with well-recognized anticancer properties (Bikle, 2016). Chronic use of CSs significantly reduces vitamin D levels (Skversky et al., 2011), possibly due to the increase in fat mass they induce (Oray et al., 2016) and the subsequent sequestration of vitamin D in adipose tissues following weight gain (Wortsman et al., 2000). A recent meta-analysis indicates that sufficient vitamin D levels are associated with a reduced risk of cancer-related mortality (Keum et al., 2022).

### 3.4 Microbiome: an aggravating factor?

Many cancers are generally associated with dysbiosis of the microbiome (an imbalance in gut flora biodiversity), including those affecting the airways and lungs, esophagus, gastric, small intestine, large intestine, liver, pancreas, and kidneys (see Table 2 in Martinez and Sabatier, 2025). Dysbiosis can promote tumor progression and metastases (Sevcikova et al., 2023). A recent study showed that chronic CS use significantly alters gut microbial diversity and composition (Couch et al., 2023), potentially reducing their efficacy and increasing the risk of infection (Chan et al., 2024). Beyond these disruptions, CSs have been linked to specific microbiome alterations in patients with inflammatory bowel disease (IBD) and irritable bowel syndrome (IBS) (Vich Vila et al., 2020). In IBD patients using oral CSs, an increased abundance of Methanobrevibacter smithii—a microorganism associated with enhanced caloric harvest and weight gain (Mathur et al., 2013)—was observed, which is a well-known side effect of corticosteroids (Kulkarni et al., 2022). Meanwhile, IBS patients using inhaled CSs showed an increased abundance of *Streptococcus* mutans and Bifidobacterium dentium (Vich Vila et al., 2020). A recent study showed that prenatal exposure to dexamethasone alters the composition of the gut microbiota by reducing alpha microbial diversity as well as its function (Lu et al., 2024), which may lead to long-term health consequences, such as diabetes (Greene et al., 2013). CSs, administered at the start of ICI treatment, are associated with reduced overall survival in several cancers, such as non-small cell lung cancer and melanoma. This decrease in survival appears to be linked to the impact of corticosteroids on T-cell mediated inflammation and their influence on the microbiome, thereby altering the response to ICIs (Spakowicz et al., 2020).

The microbiome disruptions induced by these treatments (CSs) raise concerns about their long-term impact on immunity and response to anticancer therapies.

**FIGURE 1:**
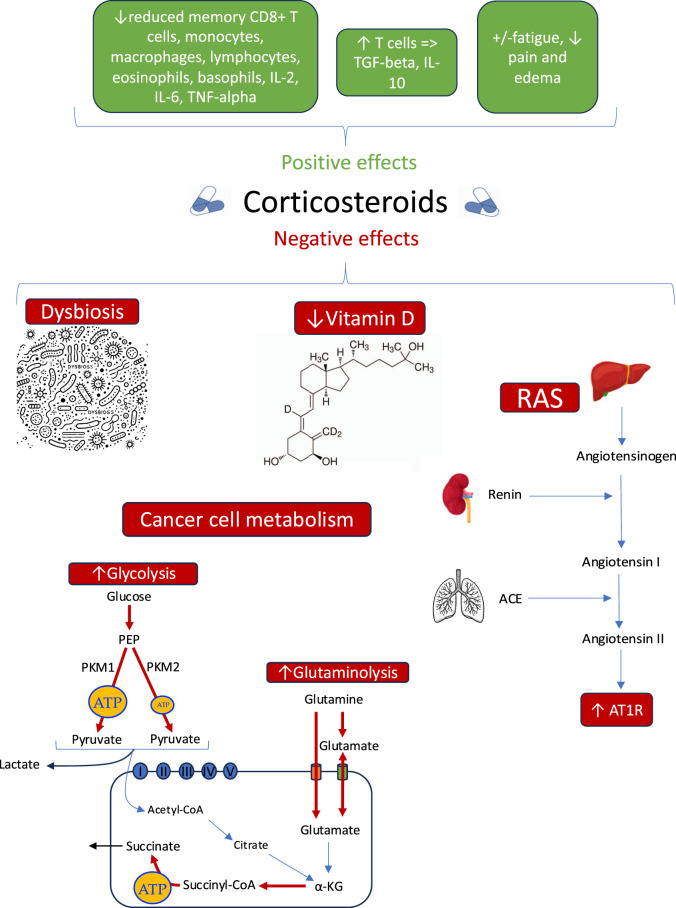
Summary of benefits and risk of corticosteroids use in cancer progression. a-KG: Alpha-ketoglutarate (cycle de Krebs), ACE: Angiotensin-converting enzyme, AT1R: Angiotensin II type 1 receptor, PEP: Phosphoenolpyruvate (glycolyse), PKM1: Pyruvate kinase M1, PKM2: Pyruvate kinase M2, RAS: Renin-angiotensin system.

The complex interplay of CSs with glucose and glutamine metabolism, the renin-angiotensin system, vitamin D, and the microbiome underscores the need for a balanced approach in oncology. These effects are summarized in Figure 1.

Given their complex effects on tumor progression, it is becoming essential to explore alternative strategies, such as combining them with metabolic modulators, RAS blockers, or microbiome-targeted interventions, which we will discuss in the next section.

## 4 Opportunities, challenges, and future directions

While CSs can be beneficial in certain cases, the potential risks highlighted in this article underscore the urgent need to explore alternative therapeutic strategies. For instance, dexamethasone, widely used to manage vasogenic edema associated with tumors or brain metastases, negatively interacts with the metabolic and molecular mechanisms previously described. In murine models, dexamethasone has been shown to reduce the effectiveness of radiotherapy by inducing p21 expression, a cell cycle inhibitor that causes cell accumulation in the G1 phase—a phase resistant to irradiation (Pitter et al., 2016). Despite its effectiveness in managing edema, dexamethasone provides no survival benefit, raising questions about its systematic use in oncology (Pitter et al., 2016). Exploring safer alternatives to CSs is essential to optimizing cancer treatment strategies. Several promising alternatives have been identified, including dietary interventions, renin-angiotensin system (RAS) blockers (sartans), and vitamin D optimization. These approaches not only address inflammation, edema, and pain—key targets of CSs—but also counteract the deleterious metabolic and molecular mechanisms associated with their use. Table 1 below summarizes the potential benefits, additional advantages, and limitations of these alternative strategies.

**TABLE 1:** Comparison of the positive effects, additional effects, and limitations of therapies compared to corticosteroid use.

Therapy		Similar effects to corticosteroids	Additionnal effects compared to corticosteroids	Limitations	Ref
Diets	Ketogenic diets	- Reduces inflammation via NF-κB inhibition and decreases cytokines (IL-1β, IL-6, TNF-α) - Anti-edematous - Alleviate pain - Reduce fatigue - Anti-tumor immunity	↓Glucose; ↑ Ketone; ↑anti-angiogenic, autophagy; pro-apoptotic effects and ↑eubiosis	- Slow effects - Compliance	Jiang and Wang (2013), Klement and Pazienza (2019), Merra et al. (2020), Duraj et al. (2024)
	Fasting	- Anti-inflammatory - Reduce fatigue - Anti-edematous	↓↓↓Glucose; ↑↑↑ Ketone; ↑ OxPhos, anti-angiogenic, autophagy, pro-apoptotic effects and eubiosis	- Requires strict monitoring - Compliance - Contraindicated in BMI <18	Jiang and Wang (2013), Klement and Pazienza (2019), Merra et al. (2020), Tiwari et al. (2022)
Pharmacological	Sartans	- Anti-inflammatory via NF-κB inhibition - Anti-edematous	↓Ang II = ↓AT1R => anti-angiogenic; ↓Glucose, IL-6, TNF-α, et IL-1β	- Slow effects - Limited efficiency in patients without associated RAS dysfunction - Drug interactions	Kitamura et al. (2007), Kourilsky et al. (2016), Hashemzehi et al. (2021)
Supplement	Vitamin D	- Anti-inflammatory (↓ROS, IL-1β, IL-6, TNF-α, NF-κB) - Decrease vasogenic edema - Reduce pain - Reduce fatigue	Partial regulation of cancer cell metabolism; ↓Renin => ↓AT1R; ↑eubiosis	- Requires prolonged supplementation - Lower efficiency in obeses	Koren et al. (2001), Dougherty et al. (2014), Liu et al. (2018), Hajimohammadebrahim-Ketabforoush et al. (2021), Helde Frankling et al. (2021), Muñoz and Grant (2022)

Ang II: Angiotensin II, AT1R: Angiotensin II, type 1 receptor, BMI: body mass index, IL-1β: Interleukin-1, beta, IL-6: Interleukin-6, NF-κB: Nuclear factor kappa-light-chain-enhancer of activated B cells, OxPhos: Oxidative Phosphorylation, RAS: Renin-angiotensin system, ROS: reactive oxygen species, TNF-α: Tumor necrosis factor-alpha.

### 4.1 Dietary strategies

Targeting both glucose and glutamine metabolism through dietary interventions and pharmacological agents while maintaining therapeutic ketosis represents a promising avenue. Various dietary strategies, such as the strict ketogenic diet (caloric restriction), the Paleolithic diet (Cruwys et al., 2020; Duraj et al., 2024), prolonged water fasting (3–7 days) (Phillips et al., 2022), and the fast-mimicking diet (Nencioni et al., 2018), have demonstrated benefits in reducing glycolysis pathway activity in cancer. For patients who cannot fast due to a BMI <18, modified fasting approaches provide viable alternatives. A 5-day cyclic fasting-mimicking diet, when combined with standard cancer therapies, has been demonstrated to enhance systemic metabolism and bolster antitumor immunity (Vernieri et al., 2022). Fasting also stimulates autophagy, which may protect normal cells while rendering cancer cells more vulnerable to treatment by modulating stress-related gene expression (p21, p16, and p53) (Erlangga et al., 2023). A meta-analysis in mice suggest that ketogenic diets alone can prolong survival and slow tumor progression compared to carbohydrate-rich diets (Klement et al., 2016). In clinical settings, a small observational study on glioblastoma patients found that adherence to a ketogenic diet for more than 6 months was associated with a significantly higher 3-year survival rate (66.7% vs. 8.3%, p = 0.0114), suggesting a potential impact on patient outcomes (Kiryttopoulos et al., 2025). These metabolic interventions exert anti-angiogenic, anti-edematous, anti-inflammatory, and pro-apoptotic effects (Jiang and Wang, 2013). Additionally, fasting, the Mediterranean diet, the ketogenic diet, and the Paleolithic diet contribute to restoring microbiota eubiosis (a balanced and healthy state of the microbiome), enhancing microbial diversity, and strengthening gut barrier function (Klement and Pazienza, 2019; Merra et al., 2020). Eubiosis contributes to reducing inflammation by promoting gut barrier homeostasis, decreasing angiogenesis, regulating epigenetic processes, and reducing metastasis (Devoy et al., 2022). In colorectal cancer models, the ketogenic diet induces shifts in the gut microbiota, increasing microbial production of stearic acid. This metabolite, along with others, exhibits pro-apoptotic effects, reducing tumor growth by inducing apoptosis in cancer cells. These benefits persist even after microbiome transplantation into germ-free mice, suggesting a causal role of the microbiota in mediating the diet’s anticancer effects (Tsenkova et al., 2025). Weight loss is also associated with the regulation of the microbiome (Koutoukidis et al., 2022). For these dietary approaches to be effective, achieving therapeutic levels of ketosis is critical. This can be monitored using the glucose/ketone index (GKI), with optimal therapeutic levels characterized by glycemia below 5 mM and ketonemia above 1–2 mM, maintaining GKI values below 2.0, ideally near 1.0 (Meidenbauer et al., 2015; Duraj et al., 2024). A combination diet-drug called Ketogenic Metabolic Therapy, may further enhance these effects. Mebendazole, for instance, inhibits glycolysis and glutaminolysis (specifically Glutaminase C for glutaminolysis) (Mukherjee et al., 2023), while 6-diazo-5-oxo-L-norleucine (DON), a pan-glutaminase inhibitor, exhibits potent antitumor activity (Olsen et al., 2015) by simultaneously inhibits glycolysis and glutaminolysis pathways (Leone et al., 2019). While acute lymphoblastic leukemia (ALL) remains one of the few cancers where CSs provide substantial therapeutic benefit, resistance mechanisms involving increased glycolytic activity (Olivas-Aguirre et al., 2021) further support the need for metabolic-targeting strategies in cancer therapy.

### 4.2 Sartans: RAS inhibitors

Another promising option involves the use of sartans, inhibitors of the RAS. Sartans can inhibit tumor growth and progression, invasion, and metastasis in many cell lines and animal model systems (see Table 2 of (Roth et al., 2019)). A recent study showed that the use of sartans improves 5-year overall survival in Nasopharyngeal carcinoma (Lin et al., 2021). A case report also demonstrated that irbesartan, led to a nearly complete radiological improvement and a significant reduction in Carcinoembryonic Antigen levels in a patient with colorectal cancer (Jones et al., 2016). Indeed, sartans can reduce vasogenic edema (Kourilsky et al., 2016), decrease blood glucose levels (Kitamura et al., 2007), and counteract pro-tumor effects linked to ACE, Ang II, and the overexpression of AT1R (Almutlaq et al., 2021). Sartans reduce inflammation by blocking the AT1R receptor, which decreases the production of pro-inflammatory cytokines such as IL-6, TNF-α, and IL-1β, (Hashemzehi et al., 2021), which are often involved in chronic inflammatory processes and the tumor microenvironment.

### 4.3 Vitamin D

Vitamin D emerges as another viable alternative. It inhibits renin, thereby lowering ACE levels, Ang II, and AT1R expression (Dougherty et al., 2014). Vitamin D also partially helps to restore normal cellular metabolism (Sheeley et al., 2022; Farahmand et al., 2023) and has been shown to reduce brain edema following brain tumor surgery (Hajimohammadebrahim-Ketabforoush et al., 2021). Vitamin D has powerful effects: it decreases **ROS** (reactive oxygen species), IL-1β (interleukin-1 beta), IL-6, IL-8, IL-17A, TNF-α (tumor necrosis factor-alpha), and NF-κB (nuclear factor kappa-light-chain-enhancer of activated B cells), while increasing MKPs (mitogen-activated protein kinase phosphatases), IL-4, and IL-10 (interleukin-4 and interleukin-10) (Koren et al., 2001; Liu et al., 2018; Muñoz and Grant, 2022). Vitamin D supplementation appears to primarily reduce cancer mortality, rather than significantly impacting overall cancer incidence (Keum et al., 2022). However, it is important to consider the limitations of vitamin D, especially in obesity-related cancers, as excess fat tissue can sequester it within the body’s fat stores (Wortsman et al., 2000).

Historically, CSs have played a central role in oncology, particularly in managing symptoms and complications associated with cancer treatments. However, an increasing number of studies highlight their potential deleterious effects on survival and tumor progression. Underlying mechanisms such as increased glucose and glutamine metabolism, disruption of the renin-angiotensin system, alterations in gut microbiota, and vitamin D underscore the complex and sometimes detrimental impacts of CS use in oncology. Several studies illustrate the need to limit the use of CSs. The study by Petrelli et al. (2020) recommends avoiding or restricting their use, particularly in patients with advanced solid tumors. In standard care for glioblastoma multiforme, it is recommended to minimize steroid use, as well as their dose and duration (Petrelli et al., 2021). Moreover, prolonged CS use in oncology, especially in cancer survivors, is not recommended in the absence of a clear clinical benefit, particularly for chronic pain relief (Paice et al., 2016). Clinical trials have also shown that prolonged CS use may be unnecessary or even harmful. In a randomized study on patients with brain metastases, Vecht et al. (1994) demonstrated that a reduced dose of dexamethasone (4 mg) was as effective as a higher dose (16 mg) while limiting side effects such as muscle weakness and Cushing’s syndrome. Furthermore, a shorter duration of CSs treatment does not increase the risk of recurrence or mortality (Gupta et al., 2022), while high-dose or prolonged use is associated with a significant reduction in PFS and OS (Terao et al., 2023). Additionally, CSs do not appear to provide a significant clinical benefit in managing cancer-related fatigue, as demonstrated in a systematic review (Sandford et al., 2023). Given these findings, CSs should be used in oncology only when unavoidable (e.g., emergencies or certain lymphoproliferative cancers), at the minimum effective dose, with regular reassessment and gradual tapering whenever possible (Ryken et al., 2010).

In light of these observations, further research is imperative to explore alternatives to CSs and optimize therapeutic strategies. Promising approaches include vitamin D optimization, the use of sartans, and dietary strategies—such as Ketogenic Diets—which could, in some cases, replace CSs in combination with other treatments. The future of cancer treatment relies on a balanced, evidence-based approach to maximize therapeutic benefits while minimizing the risks associated with CS use.

## Data Availability

The original contributions presented in the study are included in the article/supplementary material, further inquiries can be directed to the corresponding authors.
